# Patterns of selective constraints in noncoding DNA of rice

**DOI:** 10.1186/1471-2148-7-208

**Published:** 2007-11-01

**Authors:** Xingyi Guo, Yu Wang, Peter D Keightley, Longjiang Fan

**Affiliations:** 1 Institute of Crop Science & Institute of Bioinformatics, Zhejiang University, Hangzhou 310029, China; 2 Institute of Evolutionary Biology, University of Edinburgh, West Mains Road, Edinburgh EH9 3JT, UK

## Abstract

**Background:**

Several studies have investigated the relationships between selective constraints in introns and their length, GC content and location within genes. To date, however, no such investigation has been done in plants. Studies of selective constraints in noncoding DNA have generally involved interspecific comparisons, under the assumption of the same selective pressures acting in each lineage. Such comparisons are limited to cases in which the noncoding sequences are not too strongly diverged so that reliable sequence alignments can be obtained. Here, we investigate selective constraints in a recent segmental duplication that includes 605 paralogous intron pairs that occurred about 7 million years ago in rice (*O. sativa*).

**Results:**

Our principal findings are: (1) intronic divergence is negatively correlated with intron length, a pattern that has previously been described in *Drosophila *and mammals; (2) there is a signature of strong purifying selection at splice control sites; (3) first introns are significantly longer and have a higher GC content than other introns; (4) the divergences of first and non-first introns are not significantly different from one another, a pattern that differs from *Drosophila *and mammals; and (5) short introns are more diverged than four-fold degenerate sites suggesting that selection reduces divergence at four-fold sites.

**Conclusion:**

Our observation of stronger selective constraints in long introns suggests that functional elements subject to purifying selection may be concentrated within long introns. Our results are consistent with the presence of strong purifying selection at splicing control sites. Selective constraints are not significantly stronger in first introns of rice, as they are in other species.

## Background

Noncoding intronic and intergenic DNA of multicellular organisms typically comprises a large fraction of their genomes. Comparative genomic studies have revealed extensive evolutionary conservation of noncoding DNA in several mammalian and other species and are beginning to reveal the extent of potentially functional noncoding DNA [1-8]. Several lines of evidence have suggested that introns harbour a variety of untranslated RNAs (for example [9]) that are involved in mRNA processing, editing and transport [2,3]. In plants, conserved noncoding sequences have been first identified in the grasses [5-7], and evidence of regulatory elements or binding sites in these noncoding sequences has been obtained [6,7]. Interestingly, in *Arabidopsis thaliana*, based on a well-documented recent genome duplication event, intragenomic conserved noncoding sequences have also been investigated, and a unique set of noncoding DNA sequences enriched for function has been uncovered [8]. The above observations indicate that at least some functional regions in introns are likely to be under the influence of natural selection in plants in general.

Selective constraint (also known as functional or evolutionary constraint) is defined here as the factor by which evolutionary divergence of a functional sequence is reduced, relative to a neutrally evolving sequence, due to the action of purifying selection [10]. Several methods for estimating of evolutionary constraints have been proposed, and applied to coding and noncoding DNA of invertebrates and mammals [11-16]. Shabalina and Kondrashov [16] proposed a method to quantify the proportion of bases that are subject to strong purifying selection by comparing the genomes of distantly related species. It is assumed that homologous segments that show significant similarity are under strong functional constraints, otherwise are evolving free from functional constraints.

Another approach to identify functional regions in the genome is to compare sequences from species showing lower levels of divergence that are far from saturation [12]. The basis of the method is to compare the relative divergence of putatively constrained segments of the genome with that of linked putatively neutrally evolving sequences. In the selectively constrained segments, nucleotides are assumed to fall into two classes: neutral, which evolve at the same rate as the neutral sequence; or strongly constrained, in which mutations are eliminated unconditionally by natural selection. Selective constraint is then the proportion of new mutations that are strongly deleterious and removed by purifying selection [11,14,15]. It should be noted that the presence of adaptive substitutions tends to lead to underestimation of constraint, since this leads to divergence of functional regions.

One difficulty in analyzing evolutionary constraints in noncoding DNA is the inference of the correct sequence alignment. If the sequence alignment method tends to miss genuine similarities, then functional elements could be miss-assigned as non-functional. This uncertainty largely arises due to the unknown pattern of indels (gaps) between the pair of sequences [12]. A solution to this problem is to compute probabilities of alternative alignments according to explicit models of indel evolution. Based on this method, MCALIGN2 has been developed to tackle the problem of aligning noncoding DNA [17].

Selective constraints of introns have recently been investigated in *Drosophila*, mammals and other animals [11-15,18]. Several patterns of nucleotide divergence, polymorphism, and selective constraints have been uncovered (described in our results and discussion section). Until recently, no such investigation has been done in plants.

The methodology chosen to study the pattern of noncoding DNA evolution heavily depends on the dataset investigated. In general, noncoding DNA sequences need to be not too far diverged, so that it is not too difficult to align them. On the other hand, sequences should not be too similar, otherwise there may be insufficient statistical power available for comparative genomics analysis. Until now, all studies of evolutionary constraints have compared different lineages, under the assumption of the same selective pressures acting on them (e.g, *Drosophila *[12,13,15], rodents [11,14] and hominids [18]). Here, we have compared intronic sequences from just one species, a dataset including 272 paralogous pairs from a recent segmental duplication in rice (*O. sativa*). The duplication event encompasses a ~3 Mb segmental pair with perfect synteny between chromosome 11 and 12 [19]. The duplication is estimated to have occurred about 7 million years ago (mya) [19-21], although an alternative date of 21 mya has also been proposed [22]. The evolutionary divergence is compatible with estimates for human-chimpanzee (5–7 mya, [23]) and members of the *Drosophila genus *(e.g., 2.5–3.4 mya between *D. melanogaster *and *D. simulans*, [24]), which have been previously used for noncoding DNA analysis [For example, [12,13,15,18]]. Their average divergences are about 0.1 between *Drosophila simulans *and *melanogaster*, about 0.01 for human-chimpanzee, whereas ours is about 0.08. The divergence of this segment is more suitable for noncoding DNA analysis than, for example, different rice subspecies, or rice and other cereals. Rice has two cultivated subspecies, *indica *and *japonica*, for which the genomes have been sequenced. However, the two subspecies separated within about 0.5 mya [25,26], so their sequence similarity is too high and power to infer constraints is low. The divergence time of rice and other cereals is estimated to be about 50 mya [27], and alignment of noncoding sequences between them is usually problematic.

## Results and Discussion

### Compilation of intron dataset

**Figure 1:**
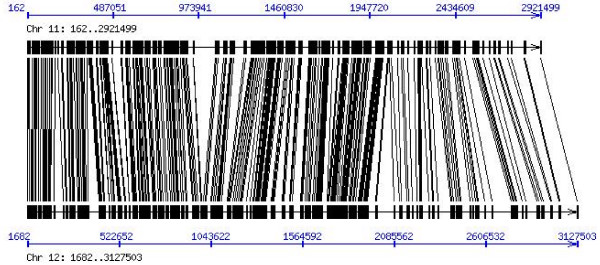
Synteny of segments from a recent duplication between chromosome 11 and 12 of rice. A total of 272 duplicate gene pairs (lines) from the duplicate segments were collected and used in this study. The physical position (bp) of the syntenic segment is based on TIGR (Release 5) see Additional file 2.

After intron alignment and some necessary masking, a dataset of 605 intron pairs (i.e., 1210 introns) was generated. The 605 pairs come from 272 duplicated gene pairs (which excluded genes that are part of a transposable element) from a recent duplication of rice chromosomes 11 and 12 (Fig. 1; A chromosomal alignment between chromosome 11 and 12 is provided in Additional file 1; a list of 272 duplicated gene pairs is provided as Additional file 2). Among the 1210 introns, median length was 122 bp (average length 232 bp; this excludes sites overlapping alignment gaps). The dataset included 85 first introns of median length 159 bp (mean length 357 bp), whereas non-first introns had median length 118 bp (mean 210 bp). It should be noted that only first intron pairs in which both introns were first introns were considered, and the same criterion was used for non-first introns. First introns are significantly longer than non-first introns (Wilcoxon two-sample test, W = 4961, *P *= 0.013), which parallels findings for other species investigated [11-15,18]. Our dataset of 272 duplicated gene pairs is similar to that investigated by Wang *et al*. [20], and other studies (such as The Rice Chromosomes 11 and 12 Sequencing Consortia [19]), although the identification approaches used by us are slightly different.

In this study, we employed several methods to minimize the frequency of incorrect alignments. These included amino acid-guided methods (see methods section) to anchor the coding regions of a paralogous gene pair (T-COFFEE), alignment using explicit models of indel evolution (MCALIGN2), and the use of two masking protocols for nonhomologous sites (for details see methods section). Our finals sample size of 605 intron pairs from 272 loci is compatible with other similar studies. For example, 200–300 loci were used by Keightley and Gaffney [11], 24 loci by Halligan *et al*. [12] and 225 intron segments by Haddrill *et al*. [13].

### Intron length

**Figure 2:**
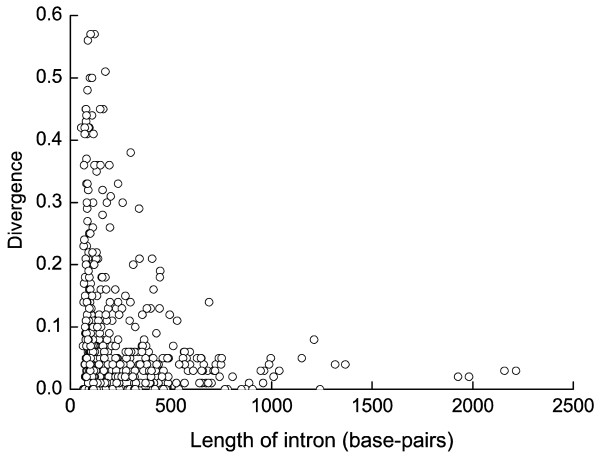
The relationship between level of divergence and intron length. There is a significant negative correlation for this data set (Spearman correlation coefficient *R*_*s *_= -0. 112, *P *= 0.006).

A negative correlation between intron length and nucleotide divergence is present in our dataset (Spearman correlation coefficient *R*_*s *_= -0.112, *P *= 0.006) (Fig. 2). This result therefore suggests that regulatory elements may be more common in long than short introns. A significant negative correlation between divergence and intron length has also been observed in other species that have been investigated (such as rodents and *Drosophila*) [11-15].

### Intron ordinal position

To further investigate the negative correlation between divergence and intron length described above, we divided our dataset into two subsets of first and non-first introns, and calculated correlation coefficients between length and divergence for each subset separately. The results indicate that the negative correlation between divergence and intron length is significant in first introns, while the test statistic for non-first introns is marginally significant (first: *R*_*s *_= -0.271, *P *= 0.012; non-first: *R*_*s *_= -0.089, *P *= 0.046). If introns are divided into two different sets according their length, there is a significant difference in divergence between short and long introns for first introns, whereas the difference is non-significant for non-first introns (Table 1). In some other taxa, first introns appear to have a higher frequency of regulatory elements [13]. It has thus been suggested that a relationship between intron size and divergence might only be expected for first introns [1]. Our results in rice seem to support this point.

**Table 1:** Divergence and GC content values for intronic sequences. Introns were divided into two classes based on their average intron length (232 bp): short introns, ≤232 bp; long introns, >232 bp. Divergence values (*K*_*i*_) are means across introns (standard errors are in parenthesis). Results of Wilcoxon two-sample test (*P*) between short and long intron (in column) and first and non-first intron (in line) are shown.

Introns	Divergence				GC Content			
		
	All	Short intron	Long intron	P	All	Short intron	Long intron	P
All		0.089 (0.006)	0.050 (0.005)	0.033		0.347 (0.003)	0.352 (0.003)	0.264
First	0.079 (0.012)	0.110 (0.018)	0.037 (0.006)	0.001	0.394(0.007)	0.396 (0.010)	0.391 (0.009)	0.705
Non-first	0.078 (0.005)	0.086 (0.006)	0.055 (0.006)	0.259	0.340 (0.002)	0.339(0.003)	0.341 (0.003)	0.544
P	0.4579	0.050	0.291		0.000	0.000	0.000	

**Figure 3:**
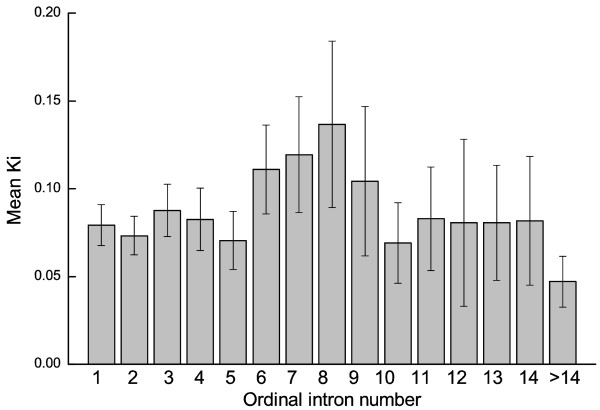
Divergence for introns of ascending ordinal position in a gene.

We further studied the relationship between the ordinal position (first, second, and so on) of introns in a gene and divergence (Fig. 3). The global correlation between intron order and *K*_*i *_is non-significant (*R*_*s *_= -0.030, *P *= 0.510). We also divided the dataset into two subsets based on first or non-first introns. Similarly, no significant difference in divergence was found between first and non-first introns (*P *= 0.458) (Table 1). This indicates that divergence does not decay slowly and regularly with the intronic ordinal position in a gene, which contrasts with the trends observed in the human-chimpanzee comparison [18].

In addition to single nucleotide mutations, we also investigate the frequency distribution of indels in first and non-first intron. A total of 1,398 indels were identified in our dataset, and no significant difference in frequencies of indel lengths between first and non-first intron was observed (non-parametric Wilcoxon test, Z = -0.052, *P *= 0.95). However, significant differences between indel numbers and lengths per base or gene pair were observed (Wilcoxon test, *P *< 0.002), with more indels in first than non-first introns. This result indicates that the evolutionary pattern of indels seems to be somewhat different from nucleotide divergence in introns in rice. Whether this trend exists in other plants or animal species need further investigation.

In summary, selective constraints seem not to be specific to first intron in rice, so our results are similar to those previously reported in *Drosophila*. In a comparison of two species of *Drosophila *(*D*. *melanogaster *and *D. yakuba*), Haddrill *et al*. [13] found that first introns evolve at similar rates to other introns. In rodents and mammals, however, it has been reported that divergence varies along introns and depend on their ordinal position within gene. Gaffney and Keightley [11] observed a negative correlation between mean intronic selective constraint and intron ordinal number in rodents, implying that first introns are more conserved other introns. Level of intronic divergence between humans and closely related species suggest that divergence also depends on intronic ordinal number [18]. The above results indicate that the rule of high constraint at first introns is not common to all taxonomic groups. Whether the phenomenon is present in other plants needs further investigation.

### Splice control sites

**Figure 4:**
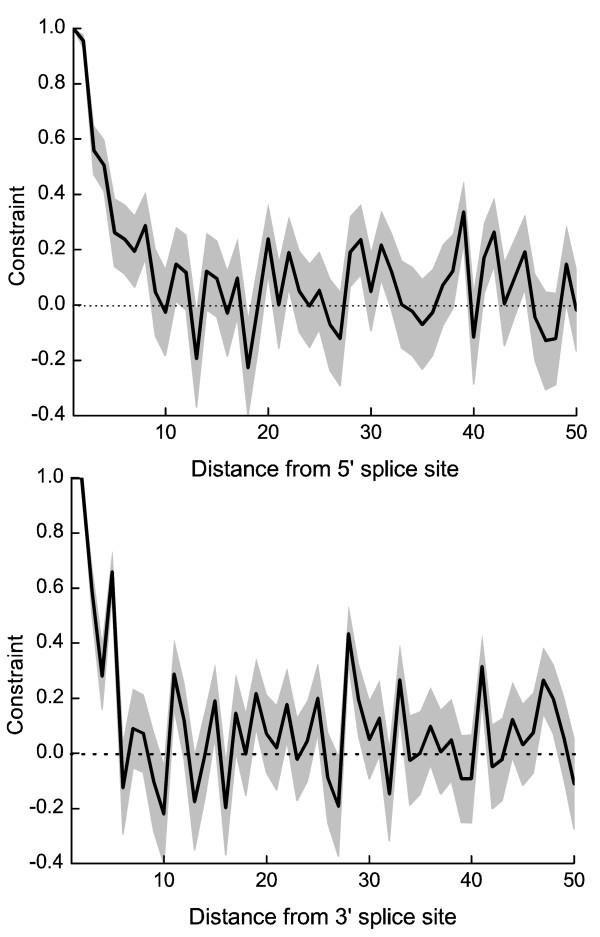
Evolutionary constraint plotted against distance from the splice sites. Gray boxes show 95% confidence intervals, estimated by bootstrapping the dataset 1,000 times.

We next examined constraints near the 5' and 3' ends of introns, which contain splice control motifs [28]. As expected, there is a strong signal of purifying selection in the sequences within 6 bp of the 5' and 3' ends, particularly at the dinucleotides adjacent to the 5' and 3' splice sites (Table 2). Similar observation has been reported in rodents [11,14] and *Drosophila *[12,15]. The distribution of constraints in introns moving away from the splice sites, however, indicates that the regions under strong constraints in rice are quite short, only about 10 bp at the 5' end and even shorter at the 3' end (Fig. 4). This situation is similar to what has been inferred in *Drosophila *[12,15].

**Table 2:** Estimates of selective constraint in intronic sequence close to the intronic splice sites. Mean (standard error) of constraint values are shown.

Intron end	Position (base pairs)	Constraint
5'		
	1–2	0.977 (0.000)
	3–6	0.394 (0.001)
	7–10	0.128 (0.001)
3'		
	1–2	1.000 (0.000)
	3–6	0.351 (0.001)
	7–16	0.007 (0.001)

### GC content

**Figure 5:**
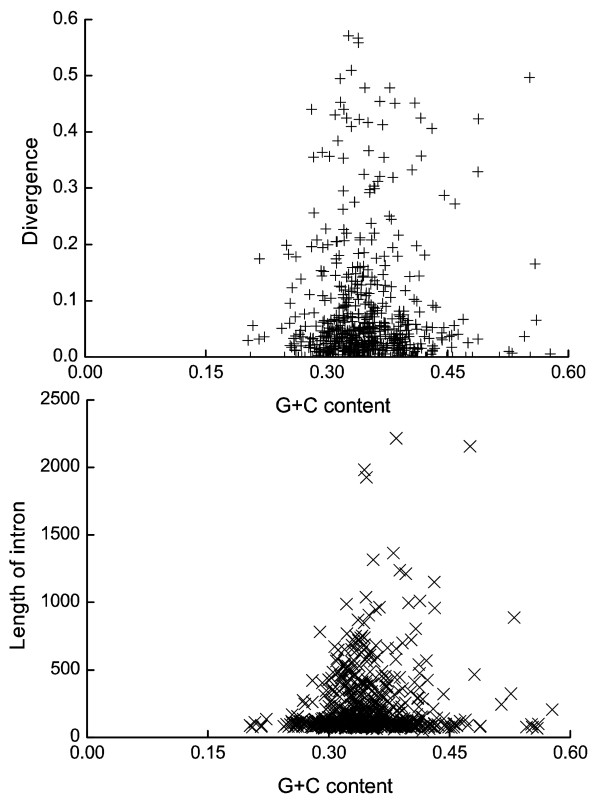
The relationship between intronic GC content and divergence and intron length. No significant relationship between them was found (divergence: *R*_*s *_= -0.019, *P *= 0.649; intron length: *R*_*s *_= 0.000, *P *= 0.993)

The base composition of *Gramineae *genes is distinct from that of dicot genes. For example, in *Gramineae *genes GC content is relative high, and there is a gradient of GC content along the direction of transcription [29]. In our previous study, we investigated GC content evolution in coding regions [30]. Here we focused on GC content evolution of intronic regions. GC content shows a significant difference between first introns and non-first introns, even in subgroups with different length (Table 1). There is also a negative gradient of GC content with intronic ordinal position, which is similar to that seen in coding sequence with transcriptional direction. These results suggest that a mechanism involving base mutation may act on first introns to elevate their GC content. Although we observed a specific pattern of nucleotide substitution in first introns (see next section), in contrast, no significant relationship between GC content and divergence (*R*_*s *_= -0.019, *P *= 0.649) or intron length (*R*_*s *_= 0.000, *P *= 0.993) was observed (Fig. 5). We also calculated the relationship between GC content and divergence and intron length in the two datasets (first and non-first intron). Similarly, no significant relationships were detected (data not shown). This result suggests that intron length and divergence are not a confounding effect of GC content in rice. In other words, GC content is dependent of the ordinal position of introns, but not divergence and length. This result is dissimilar to studies on *Drosophila *and mammals [13,18], in whichdivergence is negatively correlated with GC content. Mammalian first introns are richer in GC content and higher in divergence than other introns. In rice, first introns are also GC-rich, but do not have a significantly higher divergence than other introns.

### Substitution pattern

We used nucleotides from the fastest evolving intronic (FEI) sites as putatively neutral standards to calculate constraint. Although exonic four-fold degenerate (4-fold) sites are often used as a standard against which to test for deviations from neutrality, sites in short introns evolve faster in our data set (Table 1), so are more appropriate as a neutral standard (Table 3). The FEI sites refer to those nucleotides not close to exon boundaries (or intron splice control regions) and outside of first introns. Similar regions have previously been used to quantify functional constraints in noncoding DNA [11].

**Table 3:** Proportions of nucleotide differences at 4-fold, FEI sites and first introns. Standard errors are shown in parentheses

Sites	Type of nucleotide change					
	
	A↔C	C↔G	A↔G	T↔C	A↔T	T↔G
4-fold sites	0.0132 (0.0015)	0.0155 (0.0017)	0.0283 (0.0024)	0.0211 (0.0021)	0.0126 (0.0017)	0.0130 (0.0017)
FEI	0.0187 (0.0020)	0.0193 (0.0025)	0.0384 (0.0029)	0.0355 (0.0025)	0.0200 (0.0018)	0.0178 (0.0018)
First intron	0.0092 (0.0014)	0.0127 (0.0018)	0.0196 (0.0021)	0.0233 (0.0021)	0.0098 (0.0013)	0.0094 (0.0014)

In general, fractions of nucleotide differences at FEI sites are consistently higher than 4-fold sites and first introns. The transition events A↔G and T↔C changes are expected to be the most common substitutional changes in all categories of sites (Table 3). The situation at 4-fold sites has previously been observed in rice coding sequences, where the two changes A↔G and T↔C are predominantly from A/T to G/C, and thereby increase GC content [30]. Beside of transition T↔C, the fractions of transversion C↔G change are relatively higher than other four types of nucleotide changes in first introns compared to introns in general.

## Conclusion

We investigate selective constraints in a recent segmental duplication that includes 605 paralogous intron pairs that occurred about 7 million years ago in rice. Our observation of stronger selective constraints in long introns suggests that functional elements subject to purifying selection may be concentrated within long introns. Our results are consistent with the presence of strong purifying selection at splicing control sites. Selective constraints are not significantly stronger in first introns of rice, as they are in other species.

## Methods

### Identification of segmentally duplicated genes

Gene sequences and their annotations (release 5) were downloaded from the Rice Genome Annotation of TIGR (The Institute of Genomic research, ). The segmental duplication was identified using a reciprocal BLASTP search with E-value < 10^-14 ^within a distance of 100 kb between collinear gene pairs [31]. A total of 272 pairs of non-transposable element-derived duplicated genes were obtained between chromosomes 11 and 12. A chromosomal alignment between chromosome 11 and 12 is shown in Additional file 1 and a list of the 272 duplicated gene pairs is provided as Additional file 2.

### Identification of conserved introns and alignment masking

Following the methods of Coghlan and Wolfe [32], duplicated protein pairs were re-aligned using the T-COFFEE program [33], then used as a guide to check the quality of the alignments around the intron splice sites. An unambiguously aligned region was defined as one with at least 5 conserved amino acids and no alignment gaps in the 10 positions on each side of the splice site (20 positions in total) [34,35]. A homologous intron was identified if the location and phase were identical in the alignment of the two paralogs and if there were no other introns within 5 amino acids of this position on either side. A total of 730 pairs of intron were identified by this approach.

Intronic DNA sequences were aligned using MCALIGN2, which aligns noncoding DNA sequences based on explicit models of indel evolution [17]. To infer an appropriate indel frequency model, we first aligned the dataset with an indel model for *Drosophila *using the Jukes-Cantor model of nucleotide substitution. Then, the parameters for the alignment model (*θ *= 0.211 and *w*_1 _= 0.081) were estimated from 400 paralogous intron sequences, in which nucleotide and indel divergence are sufficiently low as to make the alignments practically unambiguous. In order to minimize the possibility of nonhomologous sites contributing to estimates of divergence, two simple masking protocols were implemented: 1) Regions that contained short aligned blocks surrounded by large gaps (>40 bp) were considered unlikely to be truly homologous and were masked off. A total of 608 pairs identified by this criteria were included for further analysis. 2) A moving window of 40 bp was used to check the degree of divergence in each alignment. Pairs containing more than 25 putatively nonparalogous sites in a window were excluded from further analyses. A total of 3 pairs was identified and excluded according to this criterion. Taken together, the final dataset used in this study contained 605 intron pairs. (Sequence alignments of the 605 intron pairs are provided as Additional file 3).

### Divergence Estimates and Calculation of Evolutionary Constraint

Introns were either analyzed as complete sequences or as partial sequences after removal of putative splice control sequences (i.e., excluding the 6 bp and 16 bp at the 5' and 3' ends of the intron, respectively). The exact limits of the control sequence are somewhat arbitrary [28]. Divergence estimates (*K*_*i*_) were generated for each alignment by applying the Jukes-Cantor correction to the number of substitution per intronic site using the distmat program from EMBOSS package [36].

In order to estimate selective constraint, a variation of the method of Kondrashow and Crow was employed, as in previous studies [11,37,38]. For each sequence, observed substitution rates were compared to that expected under neutrality. Here, we used substitution rates at FEI sites to predict expected numbers (*E*) of substitutions in adjacent intronic sequences under the assumption that point mutation rates of each possible kind are equal at FEI sites, 4-fold and adjacent intronic DNA sites. The FEI sites are defined as sequences in introns, excluding first introns and introns of length > 232 bp, and the 6 bp/16 bp at the 5'/3' end of each intron. FEIs were treated as independent observations in the data sets and were used to predict six different substitution rate parameters (A↔T, A↔C, A↔G, T↔C, T↔G, C↔G), which were calculated as the rate of substitution expected under neutrality. For each possible substitution type, Let *p*_*i *_(i = 1, 2...6) be the pairwise divergence in the FEI segment, i.e.,

*p*_*i *_= *d*_*i*_/*N*_*i*_

where *d*_*i *_is the numbers of pairwise differences of type *i*, and *N*_*i *_is the number of sites at which a change of type *i *could occur in one step (e.g., for A↔T changes, these sites are A/A, T/T and T/A). The expected number of substitutions in an adjacent interest segment is,

E=∑i=16piMi
 MathType@MTEF@5@5@+=feaafiart1ev1aaatCvAUfKttLearuWrP9MDH5MBPbIqV92AaeXatLxBI9gBaebbnrfifHhDYfgasaacPC6xNi=xI8qiVKYPFjYdHaVhbbf9v8qqaqFr0xc9vqFj0dXdbba91qpepeI8k8fiI+fsY=rqGqVepae9pg0db9vqaiVgFr0xfr=xfr=xc9adbaqaaeGacaGaaiaabeqaaeqabiWaaaGcbaGaemyrauKaeyypa0ZaaabCaeaacqWGWbaCdaWgaaWcbaGaemyAaKgabeaakiabd2eannaaBaaaleaacqWGPbqAaeqaaaqaaiabdMgaPjabg2da9iabigdaXaqaaiabiAda2aqdcqGHris5aaaa@3A60@

where *M*_*i *_is the corresponding number of intronic sites. This model assumes that symmetric mutation rates and equivalent base composition in the FEI sites and the other region of interest.

We calculated constraint by comparing E to numbers of observed substitutions (*O*):

*C *= 1 - *O*/*E*

Standard errors and confidence limits for C were calculated by bootstrapping the data values of *O *and *E *1000 times.

Proportions of difference at nucleotides in FEIs, 4-fold and intronic were treated as independent observation, respectively, and were calculated with six different substitution rate parameters (A↔T, A↔C, A↔G, T↔C, T↔G, C↔G). Standard errors and confidence for mean divergence were also calculated by bootstrapping the results by FEIs, 4-fold and intronic.

## Authors' contributions

XG and LF conceived and designed the experiments, XG and YW performed the experiments and analysed the data, XG, PDK and LF wrote the paper, and PDK advised on data analysis. All authors have read and approved the final manuscript.

## Supplementary Material

Additional file 1 Chromosomal alignment of chromosome 11 and 12 of rice. The syntenic line at left corner corresponds to the recent duplication event. The Mummer program was used with word length 80 bp. Click here for file

Additional file 2 A list of the 272 duplicated gene pairs used in this study. Locus names and their physical positions based on TIGR (Release 5) are listed. Click here for file

Additional file 3 Sequence alignments of the 605 intron pairs used in this study. Locus names and intron ordinal positions based on TIGR (Release 5) are listed. Click here for file
