# Supplementary material for: Patterns of selective constraints in noncoding DNA of rice
Source: BMC Evol Biol. 2007 Nov 1;7:208. doi: 10.1186/1471-2148-7-208 (PMC2174951; doi:10.1186/1471-2148-7-208)

Additional Data File1. Synteny of segments from the top arms of chromosome 11 and 12 were shown. Total 272 duplicate gene pairs from the duplicate segments were collected and used in this study.

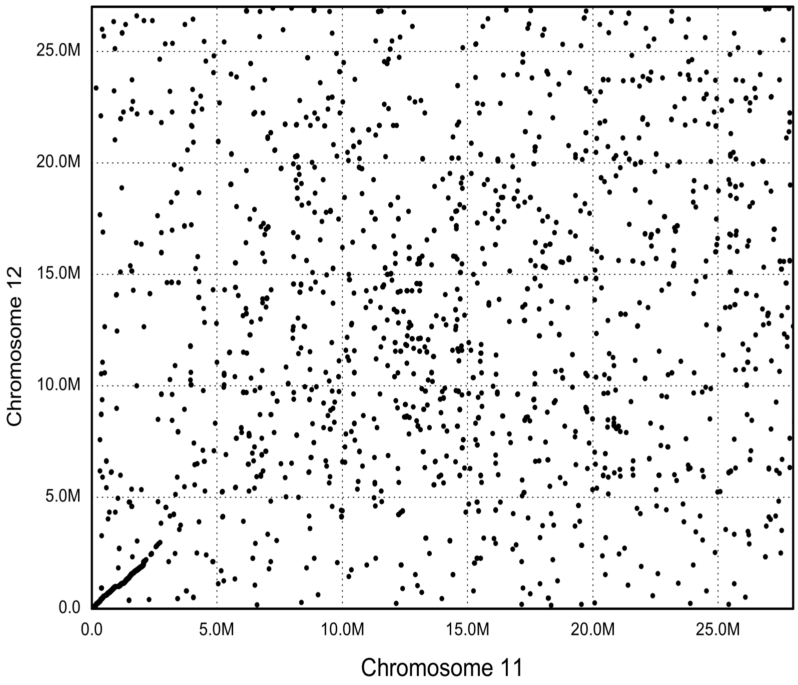

Supplement: Additional file 1 — Chromosomal alignment of chromosome 11 and 12 of rice. The syntenic line at left corner corresponds to the recent duplication event. The Mummer program was used with word length 80 bp. [file 1471-2148-7-208-S1.pdf]
